# Impact of an Ambient AI Scribe on Medical Student Objective Structured Clinical Examination Notes: Nonrandomized Clinical Trial

**DOI:** 10.2196/88264

**Published:** 2026-06-02

**Authors:** Jaideep S Talwalkar, Donald S Wright, Lee H Schwamm, Gary Leydon, Veronika Shabanova

**Affiliations:** 1 Departments of Internal Medicine and Pediatrics School of Medicine Yale University New Haven, CT United States; 2 Departments of Emergency Medicine and Biomedical Informatics and Data Science School of Medicine Yale University New Haven, CT United States; 3 Departments of Neurology and Biomedical Informatics and Data Science School of Medicine Yale University New Haven, CT United States; 4 School of Medicine Yale University New Haven, CT United States; 5 Departments of Pediatrics and Biostatistics School of Medicine Yale University New Haven, CT United States

**Keywords:** ambient scribe, artificial intelligence, AI, documentation, medical student education, note writing, objective structured clinical examination, postencounter note, standardized patient

## Abstract

**Background:**

Ambient artificial intelligence (AI) scribes for chart documentation have seen rapid adoption in clinical practice, but their educational impact on medical students has not been described.

**Objective:**

The purpose of this study was to determine the impact of an AI scribe on preclerkship medical student note writing.

**Methods:**

In this prospective nonrandomized pretest-posttest study, all first-year medical students (N=104) at a single US medical school submitted “human-only” notes based on a summative objective structured clinical examination station in May 2025. An AI scribe generated independent AI notes after the objective structured clinical examination from recorded audio. A subgroup of students (n=47) consented to complete a second “hybrid” note by revisiting their human-only notes and incorporating AI notes as perceived necessary, followed by a brief survey about the AI notes. Trained, blinded fourth-year medical student raters were randomly assigned to score all notes on 10 elements using QNOTE acceptability criteria (0=“unacceptable,” 50=“partially acceptable,” and 100=“fully acceptable”). A post hoc, exploratory element-level review was then conducted.

**Results:**

Across all elements, median evaluation scores of human-only notes were high (range 81.3-100) and similar between students who submitted “hybrid” notes and those who did not. In paired analyses between “human-only” and “hybrid” notes, the only notable element-level change was a decline in “chief complaint” scores (*P*=.05). Symptom duration was mentioned in the chief complaint section in 17% (8/47) of the AI notes. No score differences were observed in QNOTE elements requiring documentation of pertinent findings and prioritized lists. Participants agreed that the AI note “was more concise than my note” (37/47, 78.7%) and would be “helpful as a first draft” (31/47, 66%); 55.3% (26/47) agreed that the AI note “left out important details,” and 21.3% (10/47) agreed that the AI note “may reduce my ability to learn how to write a good note.”

**Conclusions:**

Interaction with AI notes among preclerkship medical students had little impact on the quality of “hybrid” notes. Chief complaint scores likely declined due to conciseness in AI notes that often omitted symptom duration. Our findings suggest that, among students who predominantly wrote close to fully acceptable “human-only” notes, there was no detriment to clinical reasoning, and students were discerning in balancing AI’s conciseness and its omissions. The lack of impact on note quality may have been due to the workflow used in this study, in which students were required to generate independent judgments before exposure to AI-generated content. Future work must explore longitudinal use of such tools using standard workflows observed in clinical settings, where AI notes serve as true first drafts. AI scribes could enhance students’ own note writing, especially for lower-performing students, although educational safeguards are necessary given the potential for harm due to overreliance on automated systems.

## Introduction

The use of ambient artificial intelligence (AI) scribes for chart documentation has seen rapid adoption among practicing physicians [1,2]. In clinical settings, AI scribes listen in the background during a patient visit and automatically create a structured medical note from the conversation [3]. Multiple recent studies have demonstrated that the use of AI scribes among practicing physicians is associated with outcomes such as reductions in time spent on documentation, cognitive load, task load, and burnout [1,4-8].

Given the degree to which documentation burden can negatively impact the graduate medical education training experience [9], Wright et al [10] conducted a pilot of AI scribes with resident physicians. Similar to the experiences of practicing physicians, residents found that AI scribes decreased task load and documentation burden.

Documentation skills are broadly taught and assessed in undergraduate medical education curricula [11-14], and the Association of American Medical Colleges lists the ability to document a clinical encounter as 1 of 13 core entrustable professional activities that medical students should be able to perform when entering residency [15]. Additionally, adult learning theory in medical education supports experiential learning, in which students engage in hands-on practice with the concepts and tools they will encounter in the workplace [16,17].

Despite the emphasis on teaching documentation skills, acceptance of the value of experiential learning in medical education, and rapid growth of AI in undergraduate medical education curricula overall [18], to our knowledge, the use of AI scribes with medical students has not been described previously.

Documentation in medical education serves not only as a communication tool but also as a cognitive exercise through which students organize data, generate differential diagnoses, and demonstrate clinical reasoning skills [19]. Introducing automation into this developmental process may have complex effects. Automation can reduce cognitive load, which may alleviate working memory constraints during demanding tasks, thereby facilitating learning and performance [20,21]. However, the cognitive load associated with tasks such as clinical reasoning is desirable in medical education for learners to develop essential skills [20].

The possibility of harm with AI scribes has led some experts to advise against early introduction of this technology in medical training [22], although the educational implications remain unclear. Shielding current medical students from technology that they will almost certainly encounter in practice may be pedagogically unsound. In the absence of empirical evidence, there is an urgent need to understand the educational effects of AI scribes. Therefore, the purpose of this study was to examine the impact of an ambient AI scribe on the quality of preclerkship medical student note writing and explore how a human-AI hybrid workflow influences note structure and content, specifically as relates to clinical reasoning.

## Methods

### Educational Setting

This prospective nonrandomized pretest-posttest study took place at a single US medical school (Yale School of Medicine, New Haven, Connecticut) in May 2025 as part of the required summative objective structured clinical examination (OSCE) that occurs at the end of the first-year Clinical Skills Course for all Yale medical students. During the OSCE, students conduct 2 complete histories and physical examinations in back-to-back 45-minute stations. Students then have 48 hours to submit a complete clinical note on their second case. All students participate in the same 2 cases in different orders, and all encounters are digitally recorded using LearningSpace (Elevate Healthcare). Passing is achieved by reaching predetermined thresholds on the history and physical examination and submitting a note. While submission of the note is required, the content and quality of the note do not factor into the OSCE score.

Prior to the study, as part of the Clinical Skills Course, students had 7 formative medical interviewing workshops with standardized patients (SPs), 10 formative physical examination workshops with peers and SPs, and 15 clinical sessions primarily with inpatients to apply these skills. Prior note-writing education and practice included a 9-minute video tutorial, a brief handout on note writing, and at least 2 previous notes on which students received narrative formative feedback from instructors.

For the May 2025 OSCE, an ambient AI scribe (Abridge Inc) was used for all students in preparation for a future classroom discussion that was not part of the study. To avoid disruption during a high-stakes assessment, the ambient AI scribe note (hereafter referred to as the “AI note”) was created using the digital recording immediately after the OSCE. Abridge-enabled mobile devices were used to create a separate AI note for each encounter using the audio extracted from each student’s original encounter. This process was previously checked for quality control in conjunction with the health system’s technology team and Abridge technical support. Quality control consisted of comparison of transcript content with source audio using multiple existing recordings within LearningSpace to ensure fidelity of transcription. Students had not used ambient AI scribes for note writing during any Clinical Skills Course sessions prior to the OSCE.

### Participants

As part of standard curricular requirements, all first-year students must complete the OSCE, including submission of a complete note. The study was described to students during an in-class announcement followed by an emailed description. Students were invited to complete a second note (described further below) as part of the study. Submission of the second note was voluntary and had no impact on OSCE grading or advancement decisions. All people involved in OSCE scoring or student advancement decisions (eg, SPs, note raters, and course faculty) were blinded regarding student participation status.

### Ethical Considerations

This study was granted approval by the Yale School of Medicine Committee to Review Student Participation in Research on January 14, 2025, and an exemption from full review due to the minimal-risk educational nature of the project by the Yale University Human Research Protection Program on January 23, 2025 (protocol ID 2000039478). Those interested in completing a second note were instructed to submit a consent form to a course administrator who was not involved in grading or advancement decisions. Participation status of students was unknown to the investigators, and data were deidentified prior to analysis. Student identity was known to the raters to facilitate assessment as the study was embedded within a curricular activity. Students who completed a second note received a US $25 meal voucher upon completion of the study.

### Note Assessment Instrument and Rater Training

Five assessment instruments were reviewed [23-27], and QNOTE [23] was selected due to alignment with the note-writing expectations of first-year medical students (ie, sufficient detail to demonstrate understanding of note structure) and the task at hand (ie, initial visit with an adult patient in an outpatient setting). As described by Burke et al [23], QNOTE consists of 12 elements: “chief complaint,” history of present illness, problem list, past medical history, medications, adverse drug reactions, social and family history, review of systems, physical findings, assessment, plan of care, and follow-up information. Each element comprises 1 to 4 components. Each component is rated as “fully acceptable,” “partially acceptable,” or “unacceptable or missing,” which correspond to numerical values of 100, 50, and 0, respectively, for the purpose of scoring. Following the methodology described in the QNOTE development, element-level scores were calculated as the means across their respective components.

Eight fourth-year medical students were recruited as raters of notes via email announcement from a course administrator. Raters received detailed instructions via email that included teaching materials about note writing that had been provided to the first-year students, 3 sample notes from the prior year’s OSCEs, and the QNOTE assessment instrument. Sample notes were chosen by the course director as examples of note quality to reflect each of the 3 overall note score rating categories described by Burke et al [23]: “fully acceptable,” “partially acceptable,” or “unacceptable.” Each of the 8 raters independently scored the 3 sample notes using QNOTE, and then they met as a group for additional rater training. The goal of rater training was to achieve good interrater reliability, defined as an intraclass correlation coefficient of at least 0.80 [28]. During a 75-minute training meeting, raters viewed their scores relative to each other and discussed their rationale and approaches to interpreting QNOTE elements. Specifically, the raters negotiated a shared mental model for “physical findings” as the OSCE required a complete physical examination, whereas QNOTE prioritized the focused, hypothesis-driven examination more commonly used in practice. Additionally, the group decided to omit 2 QNOTE elements (problem list and follow-up information) for the purposes of this study because these elements were not explicitly included in the teaching materials and typically appeared in health system notes automatically through electronic health record macros. Their exclusion was determined during rater calibration to ensure alignment between curricular expectations and assessment criteria. Therefore, the modified version of QNOTE used for this study consisted of 10 elements.

### Note Writing and Assessment Workflow

**Figure 1:**
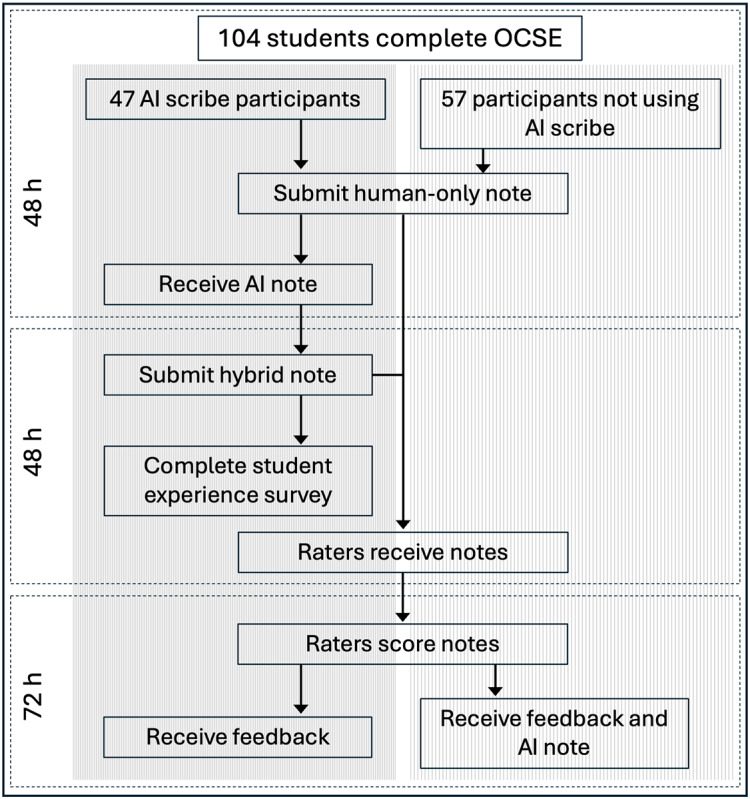
Note writing and assessment workflow. All 104 first-year medical students completed notes documenting a simulated clinical encounter. A subset of students engaged in an opportunity to modify their notes into a “hybrid note” after reviewing the output of an ambient artificial intelligence (AI) scribe. All notes were scored by blinded raters. All workflow steps were automated by an administrator to ensure blinding of student participation status and note type to raters and course faculty. OSCE: objective structured clinical examination.

All notes were requested, submitted, and assessed through a Microsoft Power Automate workflow programmed by an administrator to ensure blinding of student participation status and note type to raters and course faculty (Figure 1). Only Microsoft Word documents were permitted in the workflow. We used a cognitive forcing strategy [29] requiring students to generate notes independently before exposure to AI-generated content. Immediately following the OSCE, all 104 students received a personalized email with a link to submit their required “human-only note” based on their second case from the OSCE. Students were permitted to use any resources available to them to complete this human-only note. Upon submission of the human-only note, AI scribe participants received another personalized email with the AI note for the same case and a link to submit a “hybrid” note within 48 hours. For this hybrid note, participants were instructed to review their human-only and AI notes and submit a new note based on these documents. Thus, in a hybrid note, students would decide whether and how to update their human-only note with the information in the AI note. Upon submission of the hybrid note, raters received separate emails prompting them to score the human-only and hybrid notes using QNOTE in an electronic form.

Each note was scored once by 1 of 8 trained raters. For students who submitted both human-only and hybrid notes, the same rater scored both notes. Raters were blinded, meaning that they were unaware that the 2 notes were paired or that one represented a hybrid condition. Simultaneously, raters received the human-only notes from students who had not consented to create a hybrid note. These human-only notes were also rated via the same process. All notes were evenly distributed to raters through this automated workflow without identifiers indicating note type or participation status.

After primary scoring, we conducted a post hoc, exploratory, qualitative review of the chief complaint section of the AI notes (n=47). An unblinded investigator (JST) reviewed the text to identify recurrent patterns and to aid interpretation of the element-level results from the primary scoring. The review was descriptive and did not use a formal coding framework.

### Survey Details

Following submission of the hybrid note, participants completed an anonymous 6-item student experience survey assessing their perceptions of the AI note. Items were rated on a 5-point Likert scale ranging from “strongly disagree” to “strongly agree.” The survey was administered electronically through the previously described automated workflow.

### Statistical Analysis

An intraclass correlation coefficient for interrater reliability across all 10 QNOTE elements was calculated using a 2-way random-effects model [30,31]. Descriptive summaries of QNOTE element scores consisted of means with SDs and medians with IQRs. Comparisons of QNOTE element scores between groups (AI scribe participants vs nonparticipants) were performed using the Wilcoxon rank-sum test for independent samples. Within-group (hybrid vs human-only notes among AI scribe participants) comparisons of QNOTE element scores were implemented using Wilcoxon signed-rank tests for paired samples. A 2-sided type I error of .05 was used in statistical comparisons. We did not adjust for multiple comparisons as the statistical hypotheses corresponding to each QNOTE element did not reflect disjunction testing for a joint null hypothesis [32]. Each QNOTE element represented a distinct, prespecified construct rather than components of a single joint null hypothesis. Separate composite QNOTE scores for human-only and hybrid notes were calculated for each AI scribe participant as the mean of all 10 QNOTE elements. Composite QNOTE scores for human-only notes were then categorized into quartiles based on their empirical distribution and also plotted against their paired composite QNOTE scores for hybrid notes. Percentages of students in the lowest quartile of human-only composite QNOTE scores who improved on their hybrid notes and percentages of students whose scores declined by at least 20 points were summarized. Students’ responses to the student experience survey were presented as horizontal stacked bars of percentages for each Likert scale category. No a priori sample size or statistical power analyses were conducted as we only had access to the census of the first-year class and we did not have prior data to base our effect sizes on a power calculation. Analyses were conducted in R (version 4.5.0; R Foundation for Statistical Computing).

## Results

All 104 students submitted the required human-only write-up. A total of 45.2% (n=47) of the students consented to write a hybrid note; all completed this assignment and the student experience survey (Figure 1). Across all QNOTE elements, median evaluation scores of human-only notes were high (range 81.3-100) and were similar between students who submitted hybrid notes (n=47, 45.2%) and those who did not (n=57, 54.8%; Table 1).

**Table 1:** Comparison of QNOTE element scores on human-only and hybrid notes.

QNOTE element (0-100)			Human-only note not using AI^a^ scribe (n=57)		Participants using the AI scribe (n=47)					*P* value		
					Human-only note		Hybrid note		Unpaired samples^b^			Paired sample^c^
**Chief complaint**												
	Mean (SD)	72.8 (34.2)		69.1 (30.5)		59.6 (34.0)		—^d^			—	
	Median (IQR)	100.0 (50.0-100)		50.0 (50.0-100)		50 (50.0-100)		.41			.05^e^	
**History of present illness**												
	Mean (SD)	82.5 (19.9)		87.5 (14.3)		87.8 (14.4)		—			—	
	Median (IQR)	87.5 (75.0-100)		87.5 (87.5-100)		87.5 (87.5-100)		.21			.86	
**Past medical history**												
	Mean (SD)	82.0 (27.1)		88.6 (21.0)		87.5 (21.3)		—			—	
	Median (IQR)	87.5 (75.0-100)		100.0 (87.5-100)		87.5 (87.5-100)		.24			.34	
**Medications**												
	Mean (SD)	83.0 (27.7)		91.1 (13.4)		88.7 (20.0)		—			—	
	Median (IQR)	100.0 (83.3-100)		100.0 (83.3-100)		100.0 (83.3-100)		.35			.67	
**Adverse drug reactions**												
	Mean (SD)	87.4 (25.4)		90.8 (21.6)		86.5 (28.6)		—			—	
	Median (IQR)	100.0 (83.3-100)		100.0 (83.3-100)		100.0 (83.3-100)		.73			.17	
**Social and family history**												
	Mean (SD)	85.8 (18.6)		92.3 (8.40)		90.4 (9.55)		—			—	
	Median (IQR)	90.0 (80.0-100)		90.0 (90.0-100)		90.0 (80.0-100)		.30			.14	
**Review of systems**												
	Mean (SD)	72.5 (34.0)		79.4 (27.0)		77.3 (32.1)		—			—	
	Median (IQR)	83.3 (66.7-100)		83.3 (83.3-100)		83.3 (66.7-100)		.58			.80	
**Physical findings**												
	Mean (SD)	88.9 (21.0)		92.2 (12.0)		88.7 (19.4)		—			—	
	Median (IQR)	100 (83.3-100)		100 (83.3-100)		100.0 (83.3-100)		.77			.29	
**Assessment**												
	Mean (SD)	73.2 (28.5)		79.3 (22.5)		78.7 (21.6)		—			—	
	Median (IQR)	75.0 (62.5-100)		87.5 (75.0-100)		87.5 (68.8-100)		.42			.97	
**Plan of care**												
	Mean (SD)	77.6 (24.7)		81.9 (22.7)		76.6 (25.6)		—			—	
	Median (IQR)	87.5 (75.0-100)		87.5 (75.0-100)		87.5 (75.0-93.8)		.39			.26	

^a^AI: artificial intelligence.

^b^Comparisons of QNOTE element scores on all human-only (n=57, participants not using AI scribe) and (n=47, participants using AI scribe) write-ups using the Wilcoxon rank-sum test.

^c^Comparisons of QNOTE element scores on human-only and hybrid write-ups among AI scribe participants using the Wilcoxon signed-rank test.

^d^Not applicable as we did not compare means of individual domains due to the skewed distributions.

^e^The shift in distribution is due to fewer “fully acceptable” (16/47, 34% vs 21/47, 44.7%) and more “unacceptable” (7/47, 14.9% vs 3/47, 6.4%) hybrid notes as compared to human-only notes for the paired sample comparison of chief complaint element-level scores.

In paired analysis of QNOTE elements between human-only and hybrid notes among AI scribe participants, the largest element-level change was a decline in chief complaint scores (*P*=.05) driven by fewer “fully acceptable” (16/47, 34% vs 21/47, 44.7%) and more “unacceptable” (7/47, 14.9% vs 3/47, 6.4%) hybrid notes as compared to human-only notes, respectively. Scores on all other elements were similar (Table 1 and Multimedia Appendix 1). Post hoc analysis of AI notes revealed that symptom duration, an explicit criterion required for full QNOTE credit in the chief complaint element, was mentioned in the chief complaint section in 17% (8/47) of the AI notes.

**Figure 2:**
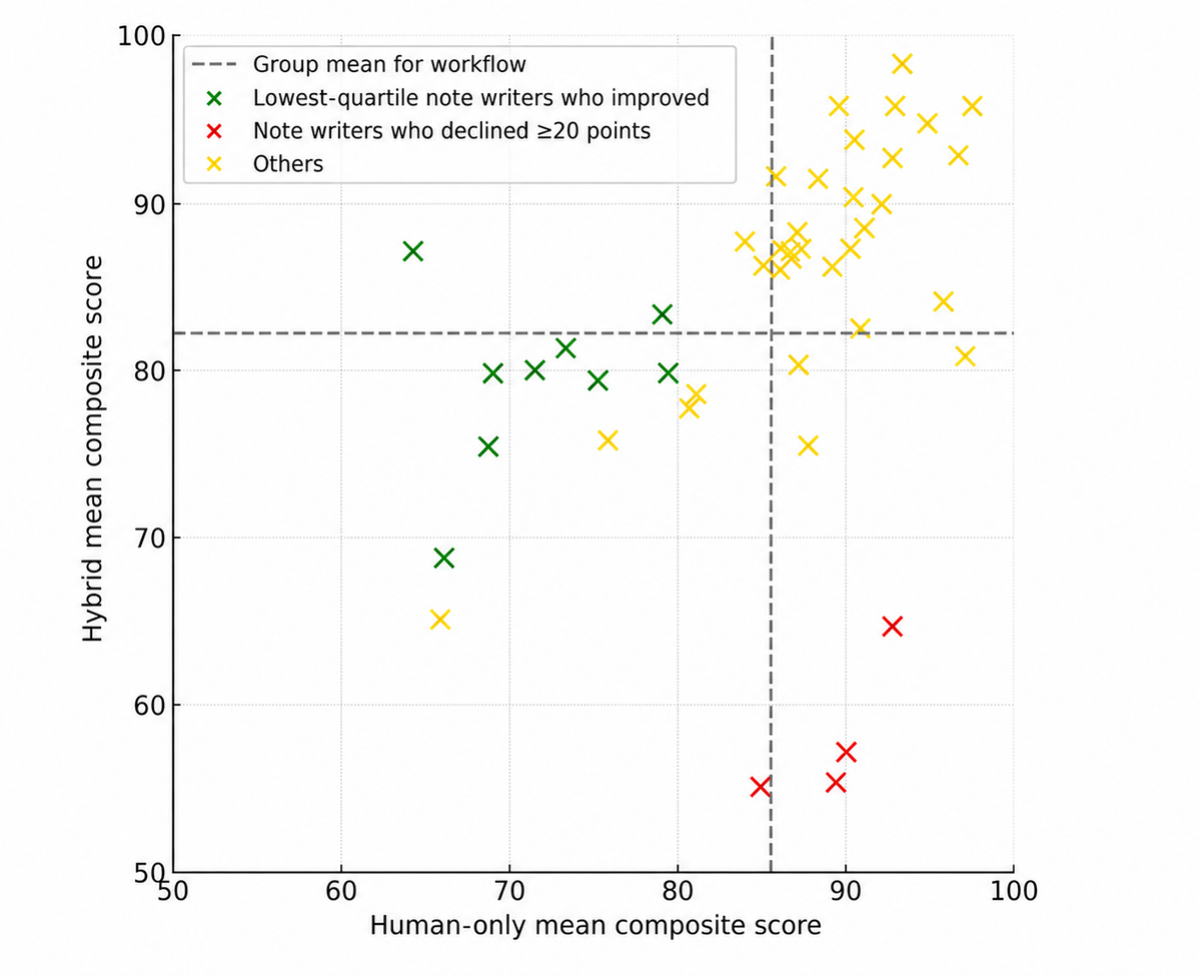
Human-only vs hybrid mean composite ordinal QNOTE scores in the paired samples (n=47). Each point represents 1 participant. Because QNOTE ratings are based on discrete ordinal categories, the plotted mean scores are quasi-continuous summary measures used for visual comparison but not for parametric inference. This figure was created with the assistance of OpenAI’s ChatGPT (GPT-5 model). The authors verified the accuracy and originality and hold ownership of the content.

Of the 12 participants in the bottom quartile of human-only composite QNOTE scores, 9 (75%) exhibited improvement with hybrid scores, whereas 8 (66.7%) in the top quartile exhibited a score decline. Overall, 10.6% (5/47) exhibited score declines of at least 20 points between human-only and hybrid notes (Figure 2).

**Figure 3:**
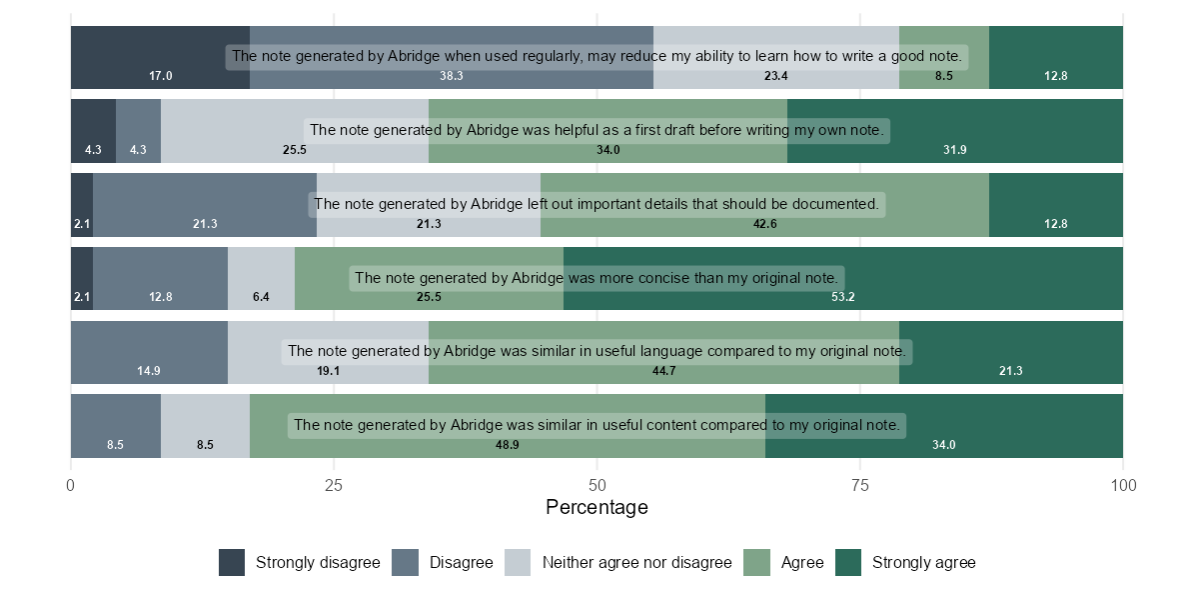
Results of the student experience survey on using an ambient artificial intelligence (AI) scribe (n=47). Students completed this 6-item survey after review of a note created by an ambient AI scribe following a simulated clinical encounter.

Participants agreed or strongly agreed that the AI write-up “was similar in useful content compared to my original note” (39/47, 83%), “was more concise than my note” (37/47, 78.7%), and would be “helpful as a first draft before writing my own note” (31/47, 66%). A total of 55.3% (26/47) agreed or strongly agreed that the AI write-up “left out important details that should be documented.” In total, 21.3% (10/47) agreed or strongly agreed that the AI write-up “when used regularly, may reduce my ability to learn how to write a good note” (Figure 3).

## Discussion

After writing high-quality “human-only” notes, interaction with notes generated by the AI scribe had little impact on the quality of the “hybrid” notes written by preclerkship medical students. Across 10 discrete note elements, the only element-level change between human-only and hybrid notes was a decline in chief complaint scores. To our knowledge, this study is the first to examine the use of ambient AI scribes for chart documentation among medical students.

Multiple prior studies have focused on the use of AI scribes among practicing physicians [1,4-8]. Tierney et al [33] and Cain et al [34] used different structured assessment instruments to demonstrate the high quality of notes after incorporation of an AI scribe in a large health system. Similarly, You et al [7] found general utility and satisfaction with AI notes in a qualitative analysis in 2 health systems. Our study suggests similar note quality with an AI workflow based on high QNOTE scores on hybrid notes and student survey responses indicating useful language and content in the AI note.

Despite these similarities with existing literature, the fundamental purpose of chart documentation for practicing physicians is different than that for medical students. In practice, the chart note serves as a communication tool between clinicians; the basis for billing, quality improvement, and performance evaluation; and a legal document [26]. While students learn about these features, they primarily write notes for educational purposes as they learn how to organize, prioritize, and synthesize information [15] and hone their ability to serve as narrators of the patient’s story [35]. Outcomes of interest in studies of AI scribes among practicing physicians, such as efficiency, cognitive load, and burnout related to note writing, are not immediately relevant to medical students. As such, in this study, we focused on students’ ability to structure a note using a conventional format while reasoning through a clinical scenario.

The QNOTE instrument emphasizes note structure with aspects of clinical reasoning embedded throughout the checklist. QNOTE elements reward clear, logical narratives that mirror a well-reasoned diagnostic process. Documentation of pertinent findings and prioritized lists are necessary for full credit on the “history of present illness” and “assessment” elements, respectively, among others [23]. As the AI scribe used in this study is designed to convert descriptions of findings into diagnostic considerations, one might expect an impact on students’ clinical reasoning. However, students on average received high scores for all elements on human-only notes with little impact after reviewing AI notes, suggesting no detriment to or augmentation of clinical reasoning overall.

The lack of impact on clinical reasoning may be due to the note writing workflow used in this study. We used a cognitive forcing strategy requiring students to generate independent judgments before exposure to potentially biasing information [29]. Cognitive forcing has been proposed as a strategy to reduce overreliance on AI [36]. In our study, requiring students to produce a human-only note prior to reviewing AI output may have preserved analytic processing and mitigated anchoring effects. While this differs from real-world clinical workflows in which AI-generated notes are the starting point, cognitive forcing through deliberate sequencing within human-AI collaboration offers a potential solution to concerns about introducing ambient AI scribes early in medical training [22].

The only observed difference between human-only and hybrid notes was a decline in “fully acceptable” scores mirrored by an increase in “unacceptable” scores in the chief complaint element. The difference in chief complaint scores was likely due to conciseness in AI notes that often omitted symptom duration, an explicit criterion for full credit on QNOTE. While errors of omission are common among AI scribes [37,38], the consistency with which duration was omitted from the chief complaint section suggests that this was due to prompt engineering [3]. In our study, over half (26/47, 55.3%) of students recognized that the AI note “left out important details that should be documented.” These omissions had minimal impact on hybrid note quality for most QNOTE elements, with scores on average approaching “fully acceptable” when written without AI. However, chief complaint was most susceptible as, on average, the scores for this element were mostly in the “partially acceptable” range among human-only notes in the first place. This decline in quality serves as a reminder of the power of automation bias, the overreliance on automated systems among early learners with AI-generated content as it can result in undesired cognitive offloading [39].

While the risk of automation bias is not unique to students, given their lack of domain expertise, early learners may be particularly vulnerable [39]. Experience and confidence in one’s own decisions decreases reliance on automated systems [40] and susceptibility to error in the face of imperfect automated outputs [41,42]. Automation bias may not be completely avoidable when confronted with complex tasks [43], but various educational safeguards have been proposed to mitigate its effect. Deliberate design of systems can help [40]. In this case, using prompt engineering better aligned with the curriculum rather than using an off-the-shelf commercial solution may have prevented the decline in chief complaint scores. However, such a solution may not be practical given resource constraints and is not a replacement for engaging students in training on the appropriate use of AI tools [22,40]. The finding that 21.3% (10/47) of the students recognized that the AI scribe “may reduce my ability to learn how to write a good note” suggests some awareness of this issue among students themselves. Our findings support the conjecture that AI tools should be released to trainees with appropriate educational safeguards and scaffolding [20,22].

The hybrid workflow may have been particularly beneficial for lower-performing students, most of whom demonstrated score increases on hybrid notes. Their improvement must be interpreted cautiously in light of potential regression to the mean as some of the highest performers saw score declines on hybrid notes. High-performing students’ human-only note scores were near the maximum possible, so ceiling compression may have limited measurable improvement and could partially explain the shift in the top quartile. As the QNOTE composite score has an upper limit, clustering near this boundary may have amplified apparent declines in the highest-performing group. It is possible that these observations may indicate true signal as they are consistent with findings in economics in which generative AI improves the quality of output for low performers while potentially having the opposite effect on high performers [44]. Establishing a causal signal will require designs that mitigate regression and ceiling effects. Future research should incorporate randomized controlled trial designs with repeated baseline measurements, longitudinal follow-up of performance trajectories, and counterbalanced AI-first vs human-first workflows (with educational safeguards) [22] to isolate the effect of AI exposure.

There are limitations to our study. This study was conducted at a single medical school within a preclerkship curriculum, limiting generalizability. Replication across multiple institutions and clerkship settings and in graduate medical education is necessary to clarify applicability longitudinally across training levels. Next, students self-selected to write hybrid notes rather than being randomly selected. However, the similar evaluation scores on human-only notes between students who submitted hybrid notes and those who did not offsets some complaint for selection bias, although unmeasured differences in motivation or attitudes toward AI may remain. Additionally, we used educational safeguards as this study took place within a summative assessment. Given the high-stakes nature of the OSCE, it was critical that the OSCE itself was unaffected by the study. Therefore, students passively received AI notes after their encounters. This workflow, which differs from ambient scribe use in workplace settings, leaves us unable to comment on the user experience with the technology. To support development of foundational skills prior to AI-supported learning [22,45], we had students write human-only notes before receiving their AI notes. This decision upheld educational best practices but represents a deviation from standard clinical workflows, which limits generalizability beyond workflows that use cognitive forcing. Finally, we did not separately analyze the AI notes because prior work has demonstrated their quality [33,34] and we had no expectation that such a note would stand alone without human review. However, future work should incorporate systematic scoring of AI notes to better understand subsection-level strengths and weaknesses to guide educational efforts.

In conclusion, the use of an ambient AI scribe following a summative OSCE for preclerkship medical students had little impact on note quality. Students found that AI notes were concise and prone to omissions and, on average, submitted high-quality notes with and without AI assistance. For low-performing students, the ambient AI scribe may have helped improve note quality. Given these findings, AI scribes could educationally enhance students’ own note writing by helping with brevity and affirming the quality of human-only notes. Enthusiasm for ambient AI scribes in medical student education should be balanced with the finding that omission of key information on an AI note can also yield lower-quality hybrid notes for elements of already lower quality without AI assistance. Our study offers a first snapshot of the impact of AI scribes on chart documentation for medical students, but future research must explore the longitudinal use of such tools. Earlier introduction could potentially jump-start skill development, although it could simultaneously trigger feelings of inadequacy [46]. Deployment later in training or with AI notes as a true first draft could increase efficiency at the expense of creating a cognitive debt due to overreliance on AI [10,22,38]. Given the rapid adoption of ambient AI scribes in clinical settings, it is crucial to further explore the educational opportunities, impact, and trade-offs of these tools.

## Data Availability

The datasets generated or analyzed during this study are not publicly available due to the need to protect student privacy, as the data were collected within a single class of students at a single medical school. The data are available from the corresponding author on reasonable request.

## Appendix

Multimedia Appendix 1 QNOTE element scores by workflow. First-year medical students (N=104) completed human-only notes after a simulated clinical encounter. A subset (n=47) then modified their notes into hybrid notes after reviewing the output of an ambient artificial intelligence scribe. Blinded raters scored 10 elements in standard clinical documentation via QNOTE. Percentages of scores in the “fully acceptable” range (76-100) on QNOTE are depicted by note type.

## Abbreviations

AI: artificial intelligence
OSCE: objective structured clinical examination
SP: standardized patient
