# Supplementary figures and images for: Impact of an Ambient AI Scribe on Medical Student Objective Structured Clinical Examination Notes: Nonrandomized Clinical Trial
Source: JMIR Med Educ. 2026 Jun 2;12:e88264. doi: 10.2196/88264 (PMC13273210; doi:10.2196/88264)

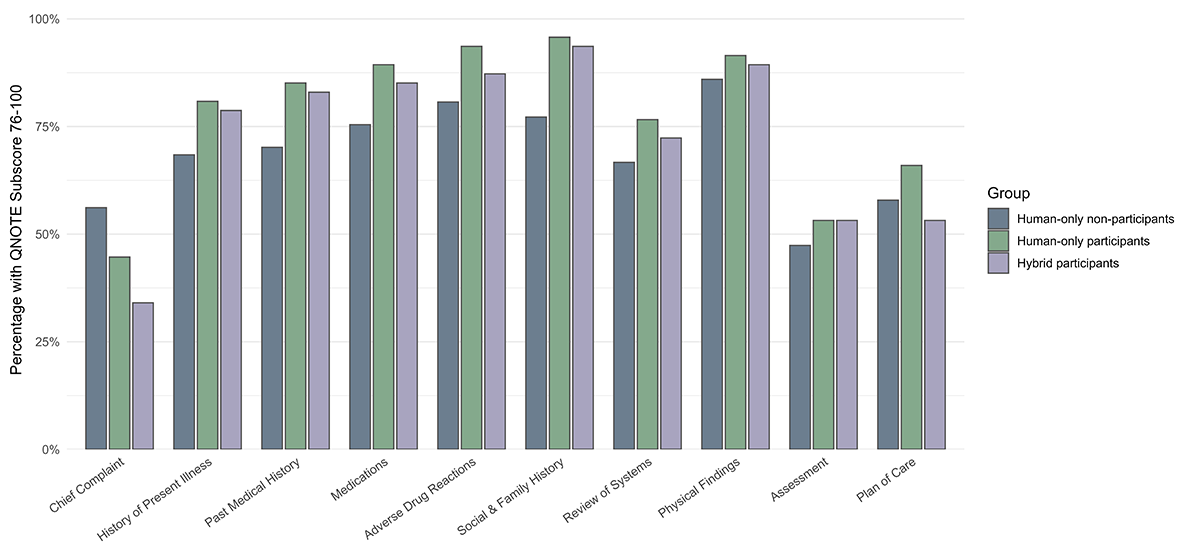

Supplement: Multimedia Appendix 1 [file mededu_v12i1e88264_app1.png]
